# Prevalence, self-awareness, and LDL cholesterol levels among patients highly suspected as familial hypercholesterolemia in a Japanese community

**DOI:** 10.1016/j.plabm.2020.e00181

**Published:** 2020-10-19

**Authors:** Hayato Tada, Junichi Shibayama, Tetsuo Nishikawa, Hirofumi Okada, Akihiro Nomura, Soichiro Usui, Kenji Sakata, Atsushi Hashiba, Akihiro Inazu, Masayuki Takamura, Masa-aki Kawashiri

**Affiliations:** a Department of Cardiovascular Medicine, Kanazawa University Graduate School of Medical Sciences, Kanazawa, Japan; b Kanazawa Medical Association, Kanazawa, Japan; c Department of Laboratory Science, Molecular Biochemistry and Molecular Biology, Graduate School of Medical Science, Kanazawa University, Kanazawa, Japan

**Keywords:** Familial hypercholesterolemia, LDL cholesterol, PCSK9

## Abstract

**Backgrounds:**

The prevalence of familial hypercholesterolemia (FH) among Japanese populations is still unclear. In addition, no prior data exist regarding the self-awareness. Accordingly, we aimed to investigate the prevalence, self-awareness, and LDL-C of patients with highly suspected as FH using data obtained in a community-based medical checkups.

**Methods:**

This study included 52,276 subjects (18,588 men, 35.6%) aged ≥40 years who underwent the Japanese specific health checkup in Kanazawa City during 2018. We assessed the self-awareness of dyslipidemia (and the age) as well as the prevalence of patients with highly suspected as FH whose naïve LDL-C levels were ≥250 ​mg/dl. Naïve LDL-C levels were estimated by the adjustment (LDL-C/0.7) for those on lipid-lowering medication. We divided subjects into 3 groups based on their naïve LDL cholesterol level (≥250 ​mg/dl, 140–249, and ≤139 ​mg/dl).

**Results:**

We identified 262 (0.5%) individuals highly suspected as FH whose naïve LDL-C levels were ≥250 ​mg/dl. Most of them (234 among 262, 89%) were under lipid-lowering medication; however, the self-awareness as dyslipidemia was not quite high (200 among 262, 76%), and their mean LDL-C level under lipid-lowering medication was 203 ​± ​35 ​mg/dl. Interestingly, the age of acknowledgement of dyslipidemia among the patients with highly suspected as FH was significantly younger than those in other categories (58 vs. 60/62 ​yrs, respectively, p ​< ​0.05 for both).

**Conclusions:**

The prevalence of patients highly suspected as FH was around 1 in 200, and their self-awareness as well as control were not still good enough among Japanese general populations.

## 1 Introduction

Familial hypercholesterolemia (FH; OMIM #143890), caused mainly by mutations in the LDL receptor, proprotein convertase subtilisin/kexin type 9 (PCSK9), or apolipoprotein B (APOB) genes, is characterized by the clinical triad of primary hyper-LDL cholesterolemia, tendon xanthomas, and premature coronary artery disease (CAD) [1], although accumulation of common single nucleotide variations and/or other LDL-associated rare genetic variations may also contribute to cause this situation [2]. The identification of patients with FH at an early stage of life could lead to their better prognosis, based on accumulated evidence of the high efficacy of LDL-lowering therapy in patients with FH [3,4]. On the other hand, the prevalence of this situation among Western countries has been estimated around 1 in 200–250 among general populations, based on several case-control studies as well as population-based studies [[5], [6], [7]]. We have previously shown that the prevalence of FH among Japanese seems to be equivalent to those reported in Western countries through an estimation via accumulated health records of hospitalized patients [8]. However, no prior data exist regarding this important issue using population-based manner.

Accordingly, we aimed to assess the prevalence of patients highly suspected as FH among the subjects who underwent specific health checkups initiated as community-based medical checkups in Japan.

## 2 Methods

### 2.1 Study subjects

A cumulative of 52,276 subjects (men ​= ​18,588, 35.6%) aged 40 or over who underwent specific health checkups in 2018 ​at Kanazawa city without missing data were included in this study. Most of the subjects visited the general practitioners to the clinics in Kanazawa city. All those data were collected and anonymized by the Kanazawa Medical Association. Details of this health checkups are described in elsewhere [[9], [10], [11]].

### 2.2 Ethical considerations

This study was approved by the Ethics Committee of Kanazawa Medical Association and Kanazawa University, and carried out in accordance with the Declaration of Helsinki (2008) of the World Medical Association. All procedures followed were in accordance with the ethical standards of the responsible committee on human experimentation (institutional and national) and with the Helsinki Declaration of 1975, as revised in 2008. Informed consents were obtained from all subjects for being included in the study.

### 2.3 Data collection in specific medical check-up

Eligible participants visited a clinic and responded to a questionnaire regarding past history of stroke, cardiac disease, kidney disease, lifestyle habits such as smoking, alcohol intake, walking, etc., and medications for hypertension, diabetes, and dyslipidemia. Measurements include the standard medical checks, such as measurement of height, weight, waist circumference, blood pressure, fasting blood glucose, hemoglobin A1c, total cholesterol, triglyceride, serum high-density lipoprotein (HDL) cholesterol (HDL-C), and low-density lipoprotein (LDL) cholesterol (LDL-C). Hypertension was defined as blood pressure ≥140/90 ​mmHg or on hypotensive medication. Diabetes was defined as the patient having 1) hemoglobin A1c ​≥ ​6.5% and blood glucose ≥126 ​at fasting, 2) hemoglobin A1c ​≥ ​6.5% and blood glucose ≥200 ​at non-fasting, or 3) hypoglycemic medication. The presence of CAD was assessed based on self-reports.

### 2.4 Adjustment of LDL-C value where any LDL-lowering medications were given

In addition to the LDL cholesterol values that were actually measured, we adjusted the LDL values for individuals on LDL-lowering medication by replacing their LDL-C values by LDL-C/0.7; this adjustment estimates the effect of statins on LDL-C values [12].

### 2.5 Evaluations

We regarded the patients with highly suspected as FH whose naïve LDL-C ≥ 250 ​mg/dl, based on the evidence showing that as much as 90% of such individuals have been the patients with FH [13]. We evaluated the prevalence of patients with highly suspected as FH, and their managements of LDL-C levels, comparing those observed in patients within other LDL-C categories. In addition, we assessed if they acknowledge their dyslipidemic status (self-awareness), and the age when they acknowledged.

### 2.6 Statistical analysis

Categorical variables were expressed as percentages. Fisher’s exact test or the chi-square test was then applied, whichever was most appropriate. Continuous variables with a normal distribution were presented as means ​± ​SD, whereas medians and interquartile ranges (IQR) were reported for values that were not normally distributed. The mean values for continuous variables were compared using the Student’s *t*-test for independent data, and median values were compared using the nonparametric Wilcoxon Mann–Whitney rank sum test or the chi-square test for categorical variables with Fisher’s post-hoc test. All statistical analyses were conducted using R statistical software version 3.4.1 (The R Project for Statistical Computing, Vienna, Austria), and *P*-values ​< ​0.05 were considered statistically significant.

## 3 Results

### 3.1 Prevalence of individuals whose naïve LDL-C level ≥250 ​mg/dl

**Fig. 1:**
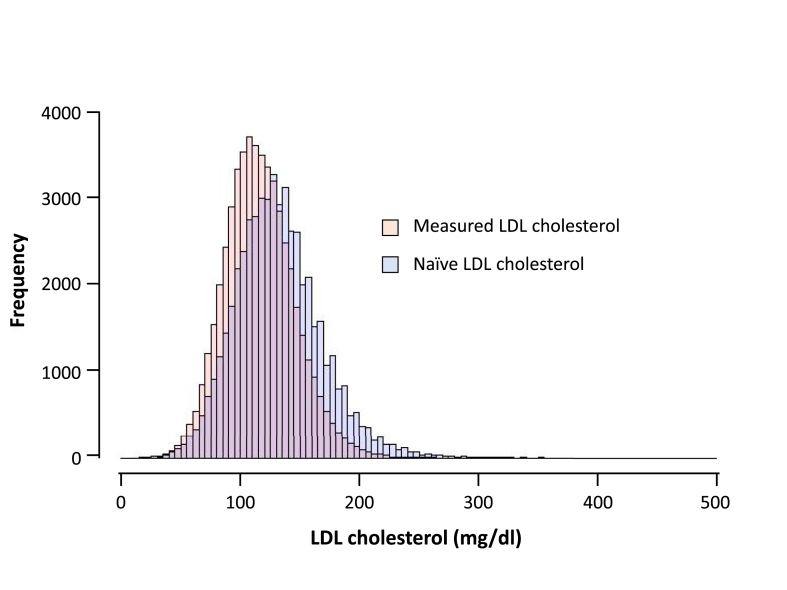
Distribution of LDL-C Light orange: measured LDL-C, Light blue: naïve LDL-C. X axis represents LDL-C (mg/dl). Y axis represents frequency. (For interpretation of the references to colour in this figure legend, the reader is referred to the Web version of this article.)

Obtained LDL-C level as well as estimated naïve LDL-C level were normally distributed in this study. (Fig. 1). Under these conditions, we identified 262 individuals (0.50%) whose naïve LDL-C level ​≥ ​250 ​mg/dl among 52,276 individuals.

### 3.2 Characteristics of study subjects

Clinical characteristics of study subjects are shown in Table 1. There was no significant differences in age among those categories. Under this condition, the proportion of male in the patients with highly suspected as FH was significantly lower than any other categories (20.6 vs. 24.6/36.7%, *p* ​< ​0.05 for both). In addition, we observed significantly less proportion of hypertension and diabetes among the patients with highly suspected as FH, compared with other categories (37.8 vs. 46.0/44.9%, *p* ​< ​0.05 for both, 10.7 vs. 13.1/12.2%, *p* ​< ​0.05 for both, respectively).

**Table 1:** Characteristics of the study subjects.

	All	Highly suspected as FH (Naïve LDL cholesterol ​≥ ​250 ​mg/dl)	Hyper LDL cholesterolemia (140 ​mg/dl ​≤ ​Naïve LDL cholesterol ​< ​250 ​mg/dl)	Normal LDL cholesterolemia (Naïve LDL cholesterol ​< ​140 ​mg/dl)
	*N* ​= ​52,276	*N* ​= ​262	*N* ​= ​4742	*N* ​= ​47,272
Age (yr)	72 ​± ​11	72 ​± ​10	72 ​± ​10	72 ​± ​11
Male (%)	18,588 (35.6)	54 (20.6)	1167 (24.6)	17,367 (36.7)
Hypertension (%)	23,485 (44.9)	99 (37.8)	2180 (46.0)	21,206 (44.9)
Diabetes (%)	6432 (12.3)	28 (10.7)	623 (13.1)	5781 (12.2)
Smoking (%)	4652 (8.9)	19 (7.3)	401 (8.5)	4232 (9.0)
Total cholesterol (mg/dl)	201 ​± ​35	292 ​± ​42	237 ​± ​31	196 ​± ​32
Triglycerides (mg/dl)	100 [72−143]	137 [99−185]	120 [88−165]	98 [70−140]
HDL cholesterol (mg/dl)	61 ​± ​16	59 ​± ​14	60 ​± ​14	61 ​± ​16
LDL cholesterol (mg/dl)	117 ​± ​29	203 ​± ​35	152 ​± ​14	112 ​± ​26
Naïve LDL cholesterol (mg/dl)	132 ​± ​36	277 ​± ​28	199 ​± ​17	124 ​± ​28
LDL-lowering therapy (%)	17,490 (33.5)	234 (89.3)	3707 (78.2)	13,549 (28.7)
Acknowledgement of dyslipidemia (%)	15,593 (29.8)	200 (76.3)	3145 (66.3)	12,248 (25.9)
Age of acknowledgement of dyslipidemia	62 ​± ​10	58 ​± ​12	60 ​± ​10	62 ​± ​10
Coronary artery disease (%)	6382 (12.2)	27 (10.3)	500 (10.5)	5855 (12.4)

FH: familial hypercholesterolemia.

### 3.3 Self-awareness of dyslipidemia, treatment rate, and control

As much as 234 (89.3%) patients with highly suspected as FH were under LDL-lowering treatments, and its percentage was significantly higher than those in other categories, and 200 (76.3%) have recognized themselves as dyslipidemia. In other words, 62 (13.7%) patients exhibiting extremely elevated LDL-C level did not recognize themselves as dyslipidemia. Their mean LDL-C level under the treatment was 203 ​mg/dl. Interestingly, the age of acknowledgement of dyslipidemia among the patients with highly suspected as FH was significantly younger than those in other categories (58 ​yr vs. 60/62 ​yrs, *P* ​< ​0.05 for both).

## 4 Discussion

Using a large dataset from the community-based specific medical check-ups, we found that 1) the prevalence of individuals whose naïve LDL-C level ≥250 ​mg/dl was around one in 200, 2) The self-awareness of their dyslipidemic state was not good enough, 3) their LDL-C levels were poorly controlled.

In 2011, we have shown that the prevalence of patients with FH could be around 1 in 208 among Japanese general populations, based on the estimation using data obtained at hospitals in the Hokuriku area of Japan. However, larger proportion of Japanese populations are treated by the general practitioners in the community, thus, it is vital for us to expand such studies to community-based settings. Since 2008, community-based medical check-ups named “specific health checkups” has been started on the basis of our aging society and the increases of lifestyle-related diseases. Specific health checkups have great advantages such as the large number of participants due to the financial support from the government, and the uniformity of the investigated items. In this study we used the dataset collected in Kanazawa city, the population of which is around 500,000.

The proportions of the subjects who exhibited CAD among the individuals highly suspected as FH was much lower than those previously described investigated in hospital-based settings [14,15]. It is reasonable to see such differences, since the subjects who are treated in hospitals should have much higher rate of CAD than those treated in clinics in communities.

It is of note that much higher proportion of individuals were under LDL-lowering therapies with poor control, and that they had acknowledged their dyslipidemic state at much earlier phase of life, compared with individuals among other categories. Those facts strongly suggest that they were not always diagnosed as FH, despite their extremely elevated LDL cholesterol level. In that sense, we cannot regard the acknowledgement of dyslipidemia in this study as the awareness of FH. Moreover, we were surprised to see that there were substantial proportion of individuals who did not acknowledge their state and without any treatments at the mean age of 72.

This study has several limitations. First, this study investigated the population living in Kanazawa area, not using nation-wide dataset, which could potentially affect the results. However, we believe that large sample size could dilute such bias. Second, detailed information regarding the lipid-lowering therapies is lacking in this study. Moreover, we estimated the naïve LDL-C using a single formula, which may not be applicable to some of the individuals. Third, we did not evaluate factors associated with FH, including Achilles tendon thickness, family history information, and genetic backgrounds. However a previous study has suggested that most (~90%) of the individuals whose LDL-C ​≥ ​250 ​mg/dl turned out to be FH. Accordingly, our estimation based on this assumption should not be so deviated from the truth. Fourth, the self-awareness of dyslipidmic state and the age at their acknowledgement were assessed based on their self-reports.

In conclusion, the prevalence of patients highly suspected as FH was around 1 in 200, and their self-awareness as well as control were not still good enough among Japanese general populations.

## Source of finding

None.

## Declaration of competing interest

None.
